# Baseline haematological and biochemical reference values for healthy male adults from Mali

**DOI:** 10.11604/pamj.2019.32.5.12797

**Published:** 2019-01-04

**Authors:** Vita Serena, Miglietta Alessandro, Maurizio, Terrazzini Nadia, Sargentini Valeria, Cella Eleonora, Bachetoni Alessandra, Dicuonzo Giordano, Angeletti Silvia, Ciccozzi Massimo, Ceccarelli Giancarlo

**Affiliations:** 1 Migrant Health Research Organisation (Mi-HeRO), Centro di Ricerca Sulla Salute delle Popolazioni Mobili e Globale, Italy; Department of Public Health and Infectious Diseases, Sapienza University of Rome, Policlinico Umberto I, Rome, Italy; 2 Units of Epidemiology and Preventive Medicine, Central Tuscany Health Authority, Florence, Italy; 3 Sanitary Bureau of Asylum Seekers Center of Castelnuovo di Porto, Rome, Italy; Auxilium Società Cooperativa Sociale, Senise (PZ), Italy; 4 School of Pharmacy and Biomolecular Sciences, University of Brighton, United Kingdom; 5 Clinical Pathology, Department of Experimental Medicine, Sapienza University of Rome, Italy; 6 Unit of Medical Statistics and Molecular Epidemiology, University Campus Bio-Medico of Rome, Italy; 7 Clinical Pathology and Microbiology Laboratory, University Hospital Campus Bio-Medico of Rome, Italy; 8 Unit of Clinical Laboratory Science, University Campus Bio-Medico of Rome, Italy

**Keywords:** Haematological reference range, Malian adults, sub-saharan migrants

## Abstract

**Introduction:**

Haematological reference values are very important for diagnostic orientation and treatment decision. The aim of this study was to establish haematological reference values for Malian healthy adults.

**Methods:**

A cross-sectional study including 161 male Malians aged between 19 and 54 years old was performed. Median and reference ranges were calculated for haematological and biochemical parameters. Parametric student's t-test was used to determine any statistically significant differences by age, smoker status, body mass index (BMI) and occupation. Ranges were further compared with those reported for other African, Afro-American and Caucasian populations.

**Results:**

Increased levels of MCV, MCH, PLT and EOS were found in younger Malians who had abnormal BMI and altered platelets parameters. Notably, significantly lower eosinophil and monocyte counts were observed in Malians compared to Europeans The smoking status did not seem to directly affect RIs.

**Conclusion:**

This is the first study to determine normal laboratory parameters in Malian adult males. Our results underscore the necessity of establishing region-specific clinical reference ranges that would allow clinicians and practitioners to manage laboratory tests, diagnosis and therapies. These data are useful not only for the management of patients in Mali, but also to support European and American clinicians in the health management of asylum seekers and migrants from Mali.

## Introduction

Since 2012, Mali has been in the grip of an unprecedented political crisis, causing a constant and numerically significant migration to Southern Europe. As a consequence of the increase in migration flows, the need for access to diagnostic tests and medical evaluations by Malians is steadily growing in host countries. Therefore, general practitioners and clinicians need more information on the health status of asylum applicants, for diagnosis orientation and treatment decisions. Reference intervals (RIs) of haematologic indices have not yet been established in Mali and in most African countries. RIs have an important role in clinical practice and are used in disease screening as well in assessing disease progression and treatment response. The use of accurate RIs can reduce the risk of disease misdiagnosis, allows an early diagnosis and improves patient care. Guidelines by the International Federation of Clinical Chemistry (IFCC) recommend that every country establish RIs of healthy adult populations [1]. Common practice in African countries is to use laboratory reference values derived from data collected from populations living in industrialized countries [2]. Discrepancies between normal reference values in industrialized and African countries have been reported in the literature [3, 4], possibly due to factors such as age, sex and geographic origin of different ethnic groups [5, 6]. The main aim of our study was to determine RIs of hematological and clinical chemistry parameters in healthy male adults coming from Mali, recruited at the Asylum Seekers Center (ASC) in Castelnuovo di Porto, Italy. The secondary objective was to evaluate the impact of anthropometric, behavioral, social and metabolic variables on haematological and biochemical analyses.

## Methods

This cross-sectional retrospective study was conducted on data from blood tests collected from adult Malians, within 3 months of their arrival in Italy, at the Internal Healthcare Facility (IHCF) of the ASC of Castelnuovo di Porto (Rome, Italy), between May and November 2014. In accordance with internal procedures, each Asylum Seeker (AS) who accessed the IHCF for the first time was interviewed using a standardized questionnaire focused on socio-demographic data and medical history. Medical staff conducted interviews with the support of mediators speaking the patient's mother tongue. Moreover, a physical examination, including the collection of vital signs and anthropometric data, was performed. The study was carried out in accordance with the Helsinki Declaration and data were collected and analyzed after receiving informed consent. Ethical approval was not required because the study was based on a retrospective analysis of data routinely collected and stored according to the Italian law on privacy (General authorization to process personal data for scientific research purposes granted by the Italian Data Protection Authority (1 March 2012 as published in Italy's Official Journal no. 72 dated 26 March 2012)).

**Reference range analysis:** Haematological and chemistry laboratory tests included: erythrocytes (RBC), haemoglobin concentration (Hb), hematocrit (HCT), mean corpuscular volume (MCV), mean corpuscular hemoglobin (MCH), mean corpuscular hemoglobin concentration (MCHC), red blood cell distribution width (RDW), concentrations of leukocytes (WBC), lymphocytes (LYM), neutrophils (NEUT), monocytes (MON), eosinophils (EOS) and basophiles (BAS), absolute number and the percentages (%) of LYM/NEUT/MON/EOS or BAS, platelets (PLT), platelet distribution width (PDW), mean platelet volume (MPV), and plateletcrit (PCT), creatinine, azotemia, glucose, aspartate aminotransferase (AST), alanine aminotransferase (ALT). Partecipantswere also tested for HbsAg, and for antibodies against HIV and HCV.

**Blood collection and testing methodologies:** Routine blood tests for hematology, blood chemistry, and virologic assessment were offered to all adults examined. For hematology, the blood was collected in a Becton Dickinson (BD) ethylene diamine tetra-acetic acid (EDTA) Vacutainer tube (Franklin Lakes, NJ, USA), while for biochemistry tests the blood was collected in a BD serum separation tube (SST) that was centrifuged (3,000 g, 3 minutes). All samples were analysed within 3 hours of collection. Blood samples were analysed using Sysmex XN 1000 automated hematology analyzer for complete blood counts, as recommended by the manufacturer. Dimension Vista 1500 Intelligent Lab System (Siemens) was used to analyze serum biochemical parameters. Hepatitis B virus surface antigen (HBsAg), hepatitis C virus antibody (HCV-Ab) and human immunodeficiency virus antibody (HIV-Ab) were detected by a third-generation enzyme linked immunoassay (Abbott).

**Statistical methods:** For each of the clinical, biochemical and haematological parameters of the study, reference values were determined at 2.5^th^-97.5^th^ percentiles; mean, median and standard deviations (±SD) were also computed. Parametric student's t-test was used to determine any statistically significant differences between the age groups ≤25 and ≥26, smokers and no smokers, normal and abnormal Body Mass Index (BMI). BMI was considered abnormal if > 25 or < 18.5 according to World Health Organization (ref http://www.euro.who.int/en/health-topics/disease-prevention/nutrition/a-healthy-lifestyle/body-mass-index-bmi) In order to test for differences in the parameters in relation to the occupation (i.e. craftsman, farmer, student/unemployed, trader), one-way ANOVA with post-hoc analysis using the Scheffe test were conducted. Analyses were carried out using Stata 10.1 software; p<0.05 values were considered statistically significant.

## Results

**Study population:** Out of 161 Malian males who had haematological and biochemical tests, 36 were excluded from the analysis due to factors that made them ineligible for study participation and could have affected their suitability for reference values. Of the 36 excluded, 25 (69.4%) were positives for HBsAg, 2(5.6%) were anti-HCV positives and 1 (2.7%) was double positive for HBsAg and anti-HCV. Moreover, 8(22.3%) partecipants were excluded because their analyses were performed 4 months after their arrival to the reception center. This left a total sample of 125 healthy participants from whom the reference ranges were calculated. Participants' median age was 25, ranging from 19 to 54 years-old (SD±6). Information on weight and height was available for 105 Malians. Most of them (n=83; 79.04%) had a normal BMI (18.75 - 24.78), 4 (3.80%) were underweight or in the mild thinness Class (i.e. BMI range: 17.28 - 18.08), 13 (12.38%) were overweight (i.e. BMI range: 25.06 - 29.73) and 2(1.90%) had an obesity class I (i.e. BMI 30.25 and 30.64 respectively). Thirty one participants (24.8%) stated to be smokers. More than half of the partecipants enrolled had no education (n=74; 63.79%). The principal occupation was farmer (n=36; 31.3%), followed by craftsman (n=29; 25.22%).

**Clinical, haematological and biochemical parameters:** The median reference values (2.5^th^-97.5^th^percentiles), mean and ±SD for clinical, haematological and biochemical parameters collected from all participants enrolled in the study are shown in Table 1. Data presented are calibrated on Malian healthy adult males, unweighted for anthropometric parameters and social factors.

**Table 1:** Clinical and biochemical parameters of the study population

Test	N°	%	Mean	SD	Median	2.5^th^	97.5^th^
SBP*	118	94.4	124	11.2	120	106	140
DBP**	118	94.4	77.2	9.1	80	60	90
HR***	116	92.8	75.9	12.6	73	58	100
**WBC (10^3/mL)**	125	100	7.1	1.8	7	4.6	10.1
**RBC (10^6/mL)**	125	100	5.2	0.4	5.2	4.6	6
**HB (gr/dl)**	125	100	15	1.0	15.1	13.4	16.6
**HCT (%)**	125	100	46.6	2.9	46.7	41.9	51
**MCV (fl)**	125	100	88.6	5.9	89	78	98
**MCH (pg)**	125	100	28.6	2.1	28.7	25.1	31.9
**MCHC (gr/dl)**	121	96.8	32.3	0.9	32.3	31.1	33.9
**RDV (%)**	121	96.8	14.2	1.3	14	12.6	16.5
**PLT (10^3/mL)**	125	100	219.6	46.3	222	152	297
**MPV (fl)**	121	96.8	9.5	3.6	9.1	7.6	11.3
**PCT (%)**	121	96.8	0.3	0.6	0.2	0.1	0.3
**PDW (fl)**	121	96.8	16.5	2.4	16.1	15.5	17.6
**LYM (%)**	125	100	51	9.3	52.2	34.9	63.2
**MON (%)**	125	100	8.7	2.2	8.7	5.1	12.3
**NEU (%)**	125	100	33.2	8.4	33.2	19.9	48.5
**EOS (%)**	125	100	6.5	6.1	4.5	1.4	17.2
**BAS (%)**	125	100	0.6	0.4	0.5	0.2	1.1
BUN**** (g/dl)	95	76	24.4	4.8	24	16	32
**Glycemia (mg/dl)**	123	98.4	91.9	11.8	90	78	111
**Creatinine (mg/dl)**	122	97.6	0.9	0.1	0.9	0.7	1.2
**AST (UI/l)**	123	98.4	23.3	6.9	22.0	15.0	35
**ALT (UI/l)**	123	98.4	19.2	8.3	17.0	9.0	35

* SBP Systolic Blood Pressure

** DBP Diastolic Blood Pressure

*** HR Hearth Rate

**** BUN Blood Urea Nitrogen

**Impact of anthropometric and social factors on clinical and laboratory parameters:** Samples were further stratified by age, BMI, smoker status and type of employment. The population was classified in 2 groups by age, young adults ≤25 years old and adults ≥26 years old, taking into account the median age (25 years). MCV/MCH/MON% and creatinine were statistically significantly higher in adults group (MCV p-value=0.01; MCH p-value=0.01; MON% p-value=0.02, creatinine p-value=0.01; data not shown), while PLT10^3 and EOS% were significantly higher in the young adults group (PLT10^3 p-value=0.01; EOS% p-value<0.01; (Annex 1). MPV, PCT, PDW and ALT were statistically significantly higher in the abnormal BMI group (MPV p-value=0.02; PCT p-value=0.02; PDW p-value=0.02; ALT p-value = 0.03; (Annex 1). No statistically significant differences were found between smoking and no-smoking groups (Annex 1) although HB, HCT and PLT 10^3 were at the limits of statistical significance (p=0.05). HB and HCT tended to be higher in no-smokers than in smokers, while PLT 10^3 were higher in smokers than in non-smokers. Participants enrolled in the study were divided in 4 groups, according to their type of employment: farmer, craftsman, trader, student/unemployed. Scheffe Post-hoc pairwise comparisons were performed among the four employment groups and statistically significant differences were found for the following parameters: MON% (p=0.03), EOS% (p=0.03), BAS% (p=0.01) and AST (p=0.01) (Annex 1). For MON%, comparisons showed noticeable differences (i) between farmers and craftsmen with significantly lower values (p=0.03) in farmers and (ii) between students/unemployed and craftsmen, with significantly lower values (p=0.03) in students/unemployed. For EOS%, comparisons indicated that the difference was statistically significant between traders and craftsmen (p=0.03) with traders showing higher values. For BAS%, the comparisons indicated that the difference was statistically significant between traders and farmers (p=0.01) with traders showing higher values. For AST, comparisons revealed statistically significant differences (i) between traders and craftsmen (p=0.01) with traders showing higher values and (ii) between traders and farmers (p=0.01), still with traders showing higher values (p= 0.01).

**Figure 1:**
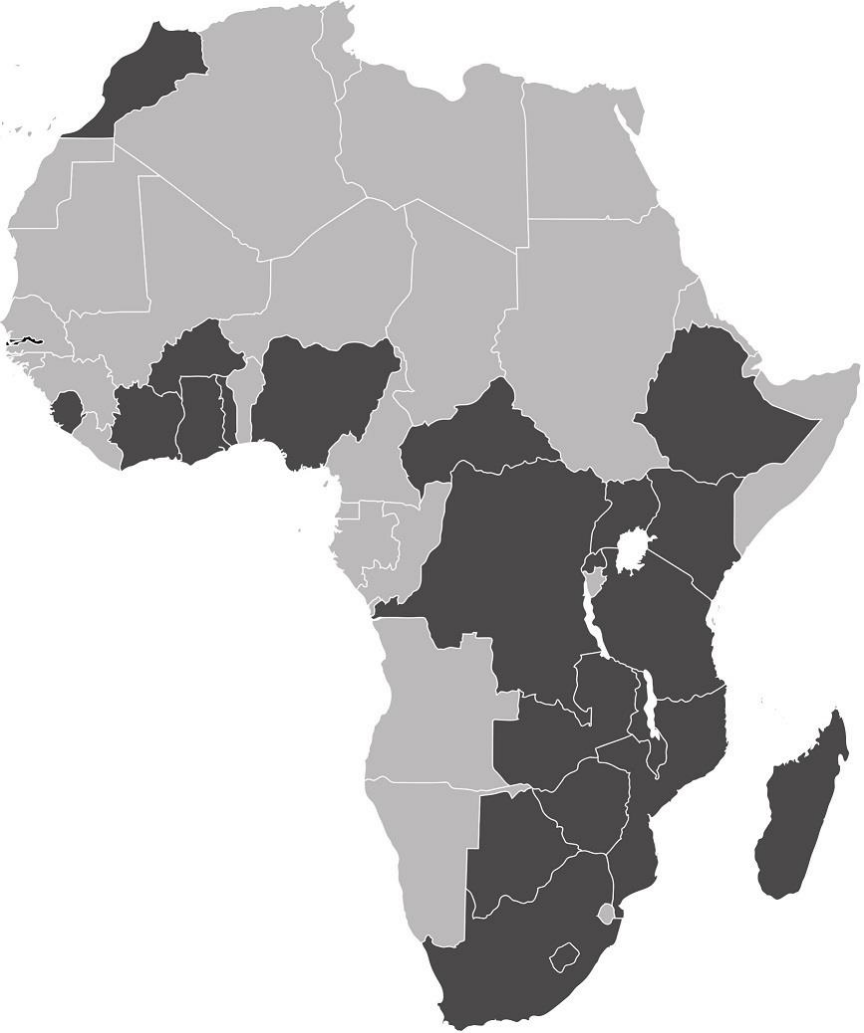
African countries where haematological reference ranges have already been described (evidenced in black)

**Figure 2:**
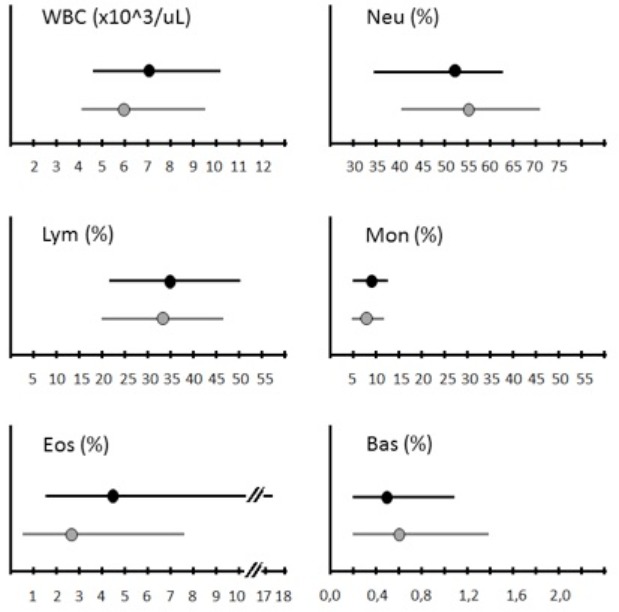
Comparison between the white cell differential count reference values for Italian (representative for South European population) and Malians participants. Data are expressed as median and reference values (2.5th-97.5th percentiles). Horizontal lines show the lower and upper limits of normal range for the population analyzed (in black Malians, in gray Italians) and the dot on each line represents the estimated median value

**Comparison between reference values in Malian males and other countries:** Table 2 and Table 2 (suite) show a summary, based on previously published data, of medians and unweighted lower and upper limits reference values (the lower limit defined as the 2.5^th^ percentile and the upper limit as the 97.5^th^percentile, according to United States National Consensus Committee on Laboratory Standards of normal ranges for laboratory tests [7]), stratified by race and ethnicity [5, 6, 8-17]. Although most Malian normal ranges appeared to be close to the RIs reported for other race/ethnicities, some deviated significantly from the corresponding range in different populations. Figure 1 shows all African countries, for which, to our knowledge, reference ranges have been reported in the literature [5, 6, 8-16, 18-25]. Moreover, considering that Italian RIs are representative of South Europe normal ranges, the comparison between the reference values for Italian [26] and Malians patients is illustrated in Table 3. Figure 2 shows the comparison between the white cell differential count reference values for Italians and Malians.

**Table 2:** comparison between reference values for Malian males and those from other countries

	Mali	Mozambique (Tembe et al.)	Ghana (Koram et al.)	Kenya (Kibaya et al.)	Tanzania (Saathoff et al.)	Botswana (Mine et al.)	Ethiopia (Yalew et al.)	CAR (Menard et al.)
**RBC (x10^6/υL)**	5.2(4.6-6)	5.1(2.7-6.1)	4.8(3.8-5.9)	5.3(4.4-6-3)	5.2(4.4-6.2)	5.18(4.2-6.3)	5.01(3.53-6.93)	5.14(4.5-6.1)
**HB (gr/dl)**	15.1(13.4-16.6)	14.1(12.3-16.4)	11.7(11.7-16.5)	9.9(8.3-11.3)	15.4(13.7-17.7)	15.3(11.9-17.1)	14.2(11.5-18)	14.9(12.3-17.3)
**HCT (%)**	46.7(41.9-51)	42.8(25.2-50.4)	44.1(37.1-51.4)	47(40-50)	46.6(40.2-53.7)	43.3(36.1-49.3)	46.9(36.2-58.6)	45(29-52)
**MCV (fl)**	89(78-98)	85.8(72.4-92.9)	NA	88(71.4-98.2)	89.3(76.4-98.8)	84(73.8-95.6)	92(85-100)*	NA
**MCH (pg)**	28.7(25.1-31.9)	NA	NA	NA	NA	29.5(23.4-33.3)	29(26.6-33-3)	NA
**PLT (x10^3/υL)**	222(152-297)	231(116-392)	209(97-356)	218(115-366)	224(147-356)	277.5(141-494)	264(128-432)*	225(124-378)
**WBC (x10^3/υL)**	7(4.6-10.1)	4.6(2.0-7.7)	5.6(3.4-8-8)	4.3(2.7-7.5)	4.4(2.8-7.9)	4.84(2.9-7.9)	5.1(3.2-8.8)*	5.05(2.09-8.3)
**NEU (%)**	33.2(19.9-48.5)	52.5(34.4-70.8)	40.5(24.4-60.7)	42(20-70)	47.3(31.7-69.3)	52.5(29.4-73-4)	53(36-69)*	NA
**LYM (%)**	52.2(34.9-63.2)	39.1(15.5-57.1)	48.2(27.5-66.5)	45(20-60)	40.8(20.8-57.3)	8.42(15.8-56.7)	38(22-55)*	NA
**MON (%)**	8.7(5.1-12.3)	NA	NA	7(3-12)	NA	7.9(3.2-15.9)	NA	NA
**EOS (%)**	4.5(1.4-17.2)	NA	NA	4(1-20)	NA	2(0.2-12.14)	NA	NA
**BAS (%)**	0.5(0.2-1.1)	NA	NA	0(0-2)	NA	0.4(0-1.5)	NA	NA
**Gly (mg/dl)**	90(78-111)	79.2(55.8-102.7)	NA	73.8(55- 100.9)	73(53 -94)	NA	NA	NA
**BUN (mg/dl)**	24(16-32)	10.6(5-16.2)	12. 3(4.7-20.1)	7.8(4.2-12.8)	7.5(4.2 - 13.9)	NA	NA	NA
**Creatinine (mg/dl)**	0.9(07-1.2)	0.91(0.65- 1.23)	1.19(0.92- 1.59)	0.87(0.7-1.19)	0.7(0.54-1.08)	NA	NA	NA
**AST (UI/L)**	22(15-35)	25.7(16.8-45.5)	31.3(18.7 - 65)	23.9(14.9-45.3)	22.8(14.2-48.1)	NA	NA	NA
**ALT (UI/L)**	17(9-35)	15.9(6.5-53.2)	24.3(11.6- 53.1)	22.3(10.8-53.9)	19(7.7-48.3)	NA	NA	NA

°Central Africa Republic

* Values for female and males

**Table 2 (suite):** comparison between reference values for Malian males and those from other countries

	Rwanda(Karita et al.)	Uganda, Masaka (Karita et al.)	Zambia (Karita et al.)	Togo (Kueviakoe et al.)	South Africa (Lawrie et al.)	Zimbawe (Samaneka et al.)	African-American (Lim et al.)	White(Lim et al.)
**RBC(x10^6/υL)**	5.16(4.25-6.16)	5.13(3.8-6.23)	4.83(3.82-5.77)	5(3.3-6.4)	NA(3.93-4.5)	5.5(3.9-5.9)	NA(3.9-4.7)	NA(4.1-5.6)
**HB (gr/dl)**	15.8(13.2-17.7)	14.9(11.7-17.2)	14.6(12.9-16.8)	15.1(10-18.4)	NA(13.4-17.4)	15.9(10.2-15.9)	NA(12-16.4)	NA(13.4-17.3)
**HCT (%)**	45.6(39.4-51.7)	43.7(34.6-50.2)	43.55(38.1-49.4)	42.8(28-54)	NA(39-51)	48.5(33.9-48.7)	NA(36.1-49.6)	NA(38.7-50)
**MCV (fl)**	89(78-99)	86(70-95)	91.5(75-104)	85(80-99)	NA(83.1-101.6)	88.8(68.8-100.7)	NA(74.1-99.1)	NA(82.6-99.1)
**MCH (pg)**	NA	NA	NA	29.7(26-36)	NA(27.8-34.8)	29.4(20.7-32.1)	NA(24.2-34.2)	NA(28.5-34.8)
**PLT (x10^3/υL)**	232(130-278)	192(54-351)	227.5(137-347)	236(120-443)	NA(171-388)	229(163.8-431)	NA(134-349)	NA(136-366)
**WBC (x10^3/υL)**	4.5(3-7.5)	5.3(3.1-9.6)	4.7(3.1-7.2)	41.(1.9-10.1)	3.92(10.4)	4.6(3.3-8.3)	NA(3.4-10.6)	NA(4.0-12.2)
**NEU (%)**	43.5(23-62.5)	37.1(20.7-58.7)	48.75(29.2-67)	1.6(0.5-5.4)	NA(32-76)	43.3/27.1-62)	NA(32.3-75.3)	NA(43.2- 75.3)
**LYM (%)**	42(26.4-58.6)	40.2(23.3-59.4)	39.1(22.4-59.4)	2.1(1.1-4.3)	NA(18-56)	43(28.4-59)	NA(16.8-54.2)	NA(16-43.5)
**MON (%)**	8.9(5.4-14.8)	8.5(4.9-14.8)	7.4(4.2-12.1)	0.2(0.05-0.8)	NA(4-12)	8.6(4.6-12.1)	NA(3.4-12.0)	NA(3.8-12.6)
**EOS (%)**	3.2(0.9-16.6)	8.7(1.5-28.9)	2.9(0.8-16.7)	0.2(0-0.5)	NA(0-8)	2.7(0.4-10-8)	NA(0.6-9.6)	NA(0.6-7.6)
**BAS (%)**	0.8(0.4-3.2)	0.7(0.4-1.7)	0.3(0.1-0.6)	NA	NA(0-2)	0.4(0.1-1)	NA(0-3.2)	NA(0-2.7)
**Gly (mg/dl)**	NA	NA	NA	NA	NA	79.5(63.5-99)	NA(69-220)	NA(66-161)
**BUN (mg/dl)**	NA	NA	NA	NA	NA	9.8(3.9-15.4)	NA(6-20)	NA(6-21)
**Creatinine (mg/dl)**	0.83(0.46-1.26)	0.89(0.67-1.26	0.96(0.66-1.32)	NA	NA	0.9(0.5-1.1)	NA(0.73-1.45)	NA(0.70-1.27)
**AST (UI/L)**	26(17-79)	28(17-58)	26(15-94)	NA	NA	28(12-40)	NA	NA
**ALT (UI/L)**	22(9-54)	24(11-49)	25(12-62)	NA	NA	21(5-35)	NA(11-64)	NA(12-87)

°Central Africa Republic, *Values for female and males

**Table 3:** Comparison between haematological reference values for Italian (representative for South European population) and Malian males

	Italian males			Malian males		
	Median	2.5^th^	97.5^th^	Median	2.5^th^	97.5^th^
**WBC** (x10^3/uL)	6.03	4.19	9.35	7.0	4.6	10.1
**RBC** (x10^6/uL)	5.22	4.71	5.82	5.2	4.6	6
**Hb** (g/dL)	15.5	14.2	17.2	15.1	13.4	16.6
**HCT** (%)	46	43.1	51.5	46.7	41.9	51
**MCV** (fl)	88.6	81.8	95.3	89	78	98
**MCH** (pg)	29.6	27.3	32.2	28.7	25.1	31.9
**MCHC** (g/dl)	33.4	31.9	35.9	32.3	31.1	33.9
**RDV** (%)	12.9	11.9	14.4	14	12.6	16.5
**PLT** (x10^3/uL)	232	164	350	222.0	152	297
**MPV** (fl)	10.9	9.5	12.3	9.1	7.6	11.3
**PCT** (%)	0.3	0.2	0.4	0.2	0.1	0.3
**PDW** (fl)	13.6	10.8	17.3	16.1	15.5	17.6

## Discussion

The International Federation of Clinical Chemistry recommends that each country should establish their own RIs for healthy subjects [1]. RIs play a pivotal role in clinical practice and the accuracy of the values used can reduce the risk of disease misdiagnosis. In fact, about 60-70% of all clinical decisions regarding a patient's diagnosis and treatment or hospital admission and discharge are made on the basis on laboratory test results [27]. Haematological reference values have, so far, been determined for the Caucasian population [28, 29] but not yet for all other countries. Hence, RIs for analysis of haematological parameters in routinary clinical practice in Africa are generated from Caucasian adult populations living in developed countries [30]. However, as a result of clinical trials and preventive interventions for HIV, tuberculosis and malaria, in the past two decades RIs have been established in several African countries, especially in southern, east and west Africa. Unfortunately the data reported so far is rather variable, due to discrepancies in the statistical methods used. In future, it would be desirable to have more homogeneous methods and therefore comparable data. More than a million migrants and refugees crossed into Europe in 2015 and 153,842 asylum seekers landed on the Italian coasts [31]. It is important to assess the state of health of all migrants and the correct identification of RIs relevant to different races and ethnicities, would allow clinicians and practitioners to manage laboratory tests and therapies more efficiently. Since haematological reference values have never been established for Malian people, the present study aimed to evaluate the RIs for haematological and biochemical parameters in healthy Malian male adult asylum seekers (ASs) housed in the largest Italian ASC. Many factors influence haematological values such as sex, age, ethnic origin, geographic location, climate, body mass index (BMI), and genetic differences [5, 6, 32-34]. In accordance with the internal procedure, each Asylum Seeker (AS) who accessed at IHCF for the first time completed a standardized questionnaire focused on socio-demographic data and was given a physical examination, including the collection of vital signs and anthropometric data. The study group consisted exclusively of males, mainly due to the low percentage of women hosted at the ASC. Only migrants without any disease were included into the study. Most of them were in good health status, which was expected, as many studies before have reported that ASs are generally young and healthy and only present minor health problems [35-39]. Hence, because of the selection process, the assessed RIs were not influenced by sex and previous or actual, acute or chronic diseases. On the other hand, anthropometric and social factors had only a partial impact on haematological and biochemical RIs. When we analyzed the results considering the BMI, we found that in people with abnormal BMI some of the platelet parameters (MPV, PCT and RDW) deviated from RIs, probability due to the fact that those factors have been linked with an increased risk of microvascular blood flow resistance [40] and vascular mortality [41]. Regarding chronologic age, we found an increased value of MCV and MCH with aging in Malians, in both white and black people, as already reported in literature [42, 43]. In fact, it has been suggested that MCV increases gradually during early adulthood, levels off around middle adulthood,to rise again in old age. Thus, such fluctuations might be associated with differences in red blood cell survival [42].

In agreement with other studies, we found higher level of PLT and EOS in younger Malians, when compared with older partecipants, for reasons not yet fully understood [44]. Concerning the smoking-status, smokers seem to present higher level of PLT than no-smokers, probably due to an indirect effect of nicotine causing a release of platelets in peripheral circulation [45]. Surprisingly, in our analysis Hb and HCT tended to be higher in no-smokers than in smokers, unlikely the literature data [46, 47]. These observations need to be confirmed. Considering participants' work activity, statistically significant differences were found between farmers, craftsmen, traders and students/unemployed only for white cell differential count (MON%, EOS%, BAS%) and AST. These differences (especially with regard to eosinophils) could reflect an occupational exposure to alternative types of allergens or pathogens, despite the fact that, at the time of blood sampling, the patients had no symptoms or any sign of ongoing disease. When we compared the RIs of Malians with references of white Americans, African-Americans and Europeans, we observed some interesting differences. In particular, we found a distribution of normal Malian (and Kenyan) values for EOS largely different from non-Africa resident population. The high values of EOS observed in a portion of the Malian and Kenyan people, when assessed with European or American RIs, could lead the clinician to evaluate the patient as suffering from the condition of hypereosinophilia. However, considering the specific RIs for these populations, many “apparent” hypereosinophilic syndrome individuals fall within the normal ranges and their results can be considered a physiological finding. This evidence is confirmed by the observation that for up to 50% of the asymptomatic immigrants a cause for eosinophilia was never identified, despite exhaustive evaluation [48]. In clinical practice, the clinical evaluation of these patients prudentially required a critical approach. Given the epidemiology of parasitic diseases in sub-Saharan countries, in our opinion, a full examination on the possible causes of secondary hypereosinophilia is nevertheless recommended. Practitioners should be always aware of the possible impact of the use of incorrect RIs and its fallout on clinical and laboratory evaluation of subjects from different race/ethnicity. Another interesting observation was that the median value of MON tended to be higher in Malian than in European persons. Interestingly, studies showed that the mild relative monocytosis observed in West African patients may be indicative of an anti-malaria effect played by monocytes; in fact, these cells play a role in the quality of antimalarial response and may enhance the predisposition to a favourable clinical outcome [49]. Based on an adaptive-evolutionary point of view, it is possible that the greater RIs for monocytes in Malian population than in the Europeans can be somewhat linked to factors such as the one described above. According to the Clinical Laboratory Standards Institute (CLSI) Guideline recommendations, a minimum size of 120 observations from each category is needed [50] and although the sample size of our study is considered sufficient to define RIs, our research suffers many limitations and further larger studies are needed. In particular, the absence of a female sample reduces the quality of the information collected and the use of standardized questionnaire focused on socio-demographic data and medical history may have represented a source of bias. However, despite its limitations, this paper represents the first systematic collection of biochemical and haematological data from Malian healthy persons. Unlike other similar studies, the patients' state of health was not determined from the patient, but was defined in all patients by medical evaluation.

## Conclusion

In conclusion, our data provide region-specific reference values which can be used to manage Malian patients (and populations with similar ethnic characteristics residents in neighboring areas) in and outside Africa. Taking into account the natural variations in the distributions of laboratory test results among racial/ethnic groups, this work will contribute to reduce disease misdiagnosis and to improve the quality of clinical care provided to migrants in the host country.

### What is known about this topic

- Guidelines by the International Federation of Clinical Chemistry recommend that every country establish reference intervals (RIs) for health. The haematological reference values have, so far, been determined for the Caucasian population but not yet for all others countries;
- The RIS are very important for diagnostic orientation and treatment decision.

### What this study adds

- The present study provides region-specific reference values which can be used to improve diagnosis and clinical care of Sub-Saharan Africans in and outside Africa, although further studies on adult Malian living in Mali are needed to confirm our observation.

## Competing interests

The authors declare no competing interest.

## Annexes

**Annex 1**: Stratification of the study population by age, BMI, smoking and employment status
